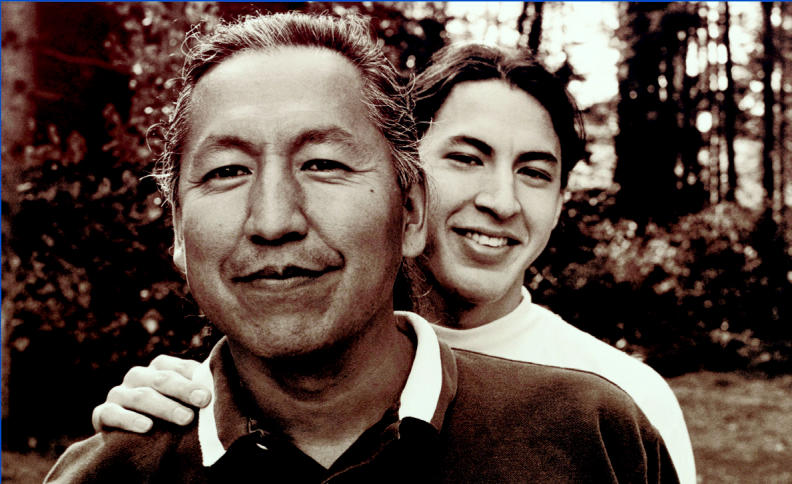# Headliners: Smoking: The Role of the Parent in Deterring Child Smoking, as Seen by Rural Native American and White Parents

**Published:** 2005-10

**Authors:** Jerry Phelps

Kegler MC, Malcoe LH. 2005. Anti-smoking socialization beliefs among rural Native American and white parents of young children. Health Educ Res 20(2):175–184.

Studies suggest that there are differences between the races in parental “anti-smoking socialization”—that is, how parents influence their children’s expectations regarding the feasibility, acceptability, and consequences of smoking cigarettes. For instance, black parents are more likely than white parents to set ground rules regarding tobacco use for their children, and are less likely to assume that teens will inevitably experiment with smoking. Now Lorraine Halinka Malcoe and NIEHS grantee Michelle C. Kegler of Emory University have compared antismoking socialization beliefs among rural white and Native American parents. Better information on how beliefs vary racially could help shape more effective ways of teaching parents to deter their children from smoking.

Teen smoking rates vary significantly between racial and ethnic groups. According to data from the Centers for Disease Control and Prevention for the year 2000, 31.8% of white high school students reported smoking in the past 30 days. Hispanic students were next at 22.6%, followed by Asian Americans at 20.6%, and blacks at 16.8%. Data on smoking among Native American teenagers are not as readily available, but some studies have indicated the rate among Native Americans overall is comparable to or higher than that of whites. In 2000, 36% of adult Native Americans smoked, compared with 24.1% of white adults.

The study showed that Native American and white parents were similar in their antismoking socialization beliefs with one exception: Native American parents were less likely to believe that schools are better than parents at teaching children about the dangers of smoking. Less educated parents were more likely to believe that strictly forbidding children to smoke only makes them want to smoke more. Consistent with earlier results, parents of both races had less stringent beliefs and a lesser sense of parental efficacy compared to black parents.

Methods to bolster antismoking socialization beliefs of less-educated parents may be important in preventing children in low-income rural communities with high smoking rates from beginning to smoke. Although limited in size and scope, this study provides evidence that future research should focus on ways to increase parental communication of antismoking beliefs and assessment of whether such interventions result in lower rates of smoking onset.

## Figures and Tables

**Figure:**